# Perceived knowledge, attitudes, and practices regarding nutrition therapy among people with prediabetes: a structural equation model analysis

**DOI:** 10.3389/fnut.2026.1952271

**Published:** 2026-10-29

**Authors:** Chengcheng Guo, Yue Yu

**Affiliations:** 1 Department of Endocrinology and Metabolism, Beijing Anzhen Hospital, Capital Medical University, Beijing, China; 2 Department of Endocrinology and Metabolism, Peking University People's Hospital, Beijing, China

**Keywords:** attitude, nutrition therapy, perceived knowledge, practice, prediabetes, structural equation modeling

## Abstract

**Objective:**

To assess nutrition therapy-related perceived knowledge, attitudes, and practices (KAP), identify associated participant characteristics, and examine the relationships proposed by the KAP framework in adults with prediabetes.

**Methods:**

This cross-sectional questionnaire study included adults with prediabetes at Beijing Anzhen Hospital between June and July 2026. Scores were categorized using modified Bloom cut-offs as low (< 60% of the maximum possible score), moderate (60–79%), or high (≥80%). Linear regression examined factors associated with KAP scores. Structural equation modeling (SEM) with weighted least squares mean- and variance-adjusted estimation evaluated relationships among three latent constructs using ordered questionnaire items.

**Results:**

A total of 449 valid questionnaires were analyzed, including 257 (57.2%) female participants. Mean scores were 12.03 ± 5.51 for perceived knowledge (possible range: 0–20), 27.21 ± 4.79 for attitude (possible range: 8–40), and 22.71±5.60 for practice (possible range: 7–35). According to modified Bloom cut-offs, low perceived knowledge (53.2%), moderate attitudes (64.8%), and moderate practices (55.7%) predominated. In SEM, perceived knowledge was positively associated with attitude (standardized β = 0.744) and practice (β = 0.226), and attitude was positively associated with practice (β = 0.469; all *P* < 0.001). The indirect association through attitude was 0.349 (95% CI: 0.257–0.444).

**Conclusions:**

Participants mainly reported limited perceived knowledge and moderate attitudes and practices. Positive relationships among the constructs may help identify potential targets for nutrition education, but measurement limitations and the cross-sectional design preclude causal or mechanistic conclusions.

## Introduction

Prediabetes is an intermediate metabolic state defined by glucose levels above normal but below the diagnostic criteria for diabetes, including impaired fasting glucose, impaired glucose tolerance, and/or elevated glycated hemoglobin levels (1). Prediabetes is highly prevalent in China. In a nationally representative survey of 173,642 Chinese adults conducted in 2018, the estimated prevalence of prediabetes was 38.1% (2). Individuals with prediabetes have a substantially increased risk of progressing to type 2 diabetes and developing cardiometabolic complications, although the magnitude of risk varies according to the underlying glycemic phenotype and other metabolic characteristics (1). Moreover, prediabetes is associated with increased risks of cardiovascular disease and mortality, highlighting the importance of early identification and preventive strategies (3).

Prediabetes represents a critical window for intervention, as lifestyle modification during this stage can substantially delay or prevent the progression to type 2 diabetes. In the US Diabetes Prevention Program, an intensive lifestyle intervention reduced diabetes incidence by 58% compared with placebo over a mean follow-up of 2.8 years (4). The Finnish Diabetes Prevention Study reported a comparable 58% reduction (5). Current clinical recommendations therefore emphasize weight management, healthy reduced-energy eating patterns, regular physical activity, and individualized medical nutrition therapy for people with prediabetes (6–8). Current consensus guidance for adults with diabetes or prediabetes defines “nutrition therapy” as the management of disease risk through modification of nutrient or whole-food intake (7). Medical nutrition therapy refers more specifically to individualized, evidence-based nutrition care delivered by qualified professionals. The term “nutrition-therapy education” is used to describe education or guidance related to these dietary strategies. Evidence from large-scale intervention studies has demonstrated that lifestyle intervention centered on dietary modification and physical activity achieved sustained weight loss and markedly reduced diabetes incidence, highlighting the effectiveness of comprehensive lifestyle strategies (9, 10). Compared with pharmacological approaches, nutrition therapy is generally considered a safe, accessible, and sustainable strategy that can be integrated into routine clinical care with minimal additional costs. However, because hyperglycemia in prediabetes is often asymptomatic, many individuals have limited awareness of their future diabetes risk and may not receive timely nutrition education or preventive support.

Nutrition therapy requires more than the provision of dietary instructions. Patients must understand why early intervention matters, accept the value and feasibility of dietary change, and translate recommendations into repeated daily decisions. Knowledge, attitude, and practice (KAP) surveys originated in the 1950s in the fields of family planning and population research and have since been widely applied to assess how individuals understand, perceive, and respond to specific health-related issues. Within the classic KAP framework, knowledge refers to awareness and understanding of relevant health information, attitude reflects beliefs, perceptions, acceptance, and motivation toward the recommended behavior, and practice represents the actual implementation of that behavior in daily life (11, 12). The core proposition of the KAP framework is that knowledge provides the cognitive basis for attitude formation, and attitudes in turn influence the adoption and implementation of corresponding practices, forming a sequential knowledge–attitude–practice pathway. These three domains are conceptually distinct because possessing knowledge does not necessarily imply a favorable attitude, and a favorable attitude does not necessarily result in corresponding practice. At the same time, they are theoretically interrelated, as knowledge may shape attitudes and attitudes may influence whether knowledge is translated into practice (11, 12). This framework has therefore been widely applied in health-related research to identify gaps between what individuals know, how they perceive recommended health behaviors, and what they actually do, thereby helping to identify potential targets for health education and behavioral intervention. For example, nutrition-related KAP research in diabetes suggests that knowledge, attitudes, socioeconomic resources, and exposure to nutrition education are associated with dietary practices, and a large multicenter survey in urban China further reported substantial deficits in medical nutrition therapy KAP among people with diabetes (13, 14). In prediabetes, where the condition is often asymptomatic and prevention relies heavily on sustained lifestyle modification, nutrition-related management requires individuals not only to understand dietary recommendations but also to recognize their importance and incorporate them into everyday behavior. The KAP framework is therefore particularly suitable for examining nutrition-related behaviors in this population. Despite this relevance, existing KAP research has predominantly focused on individuals with established diabetes, whereas individuals with prediabetes—an early and potentially reversible stage of dysglycemia—have received comparatively limited attention.

Accordingly, this study aimed to describe nutrition therapy-related KAP and its demographic and clinical correlates in adults with prediabetes, and to examine the hypothesized positive associations among perceived knowledge, attitude, and practice. Based on the KAP framework and previous empirical evidence, we hypothesized that perceived knowledge would be positively associated with attitude (H1) and practice (H2), that attitude would be positively associated with practice (H3), and that perceived knowledge would also be indirectly associated with practice through attitude (H4).

## Methods

### Study design and participants

This cross-sectional questionnaire study was conducted among adults with prediabetes at Beijing Anzhen Hospital between June and July 2026. Eligible participants were adults with prediabetes who: (1) were aged ≥18 years; (2) met the diagnostic criteria for prediabetes according to the 2026 American Diabetes Association (ADA) Standards of Care in Diabetes (1), defined as glycated hemoglobin (HbA1c) 5.7%−6.4%, fasting plasma glucose 5.6–6.9 mmol/L, and/or 2-h plasma glucose 7.8–11.0 mmol/L during a 75-g oral glucose tolerance test (OGTT); (3) were able to independently understand and complete the questionnaire; and (4) voluntarily agreed to participate and provided informed consent. Participants were excluded if they: (1) were unable to complete the survey due to severe acute illness, cognitive impairment, or psychiatric disorders; (2) were pregnant or breastfeeding; or (3) were currently participating in other dietary intervention programs or medical studies that could influence dietary behaviors. Ethical approval was granted by the Medical Ethics Committee of Beijing Anzhen Hospital (Approval No. 2026214x), and informed consent was obtained from all participants before participation in the study.

### Questionnaire

The initial questionnaire was developed with reference to relevant guidance, including the *Intervention for adults with pre-diabetes: a Chinese expert consensus (2023 edition)* and the *Nutrition Therapy for Adults With Diabetes or Prediabetes: A Consensus Report* (7), and related KAP research (13, 14). Its content was then reviewed by two endocrinologists more than 20 years of professional experience. According to their suggestions, the wording was revised to reduce duplication, ambiguity, and unnecessary technical terminology. Pilot testing was conducted among 34 individuals with prediabetes who met the same eligibility criteria as the main-study participants, to evaluate questionnaire comprehensibility, wording clarity, and preliminary internal consistency. During the pilot survey, participants were asked to provide feedback on the clarity and comprehensibility of the questionnaire items, and none reported difficulty understanding the wording or response options. In the pilot sample, the overall questionnaire demonstrated good internal consistency, with a Cronbach's α coefficient of 0.908. In the main sample, internal consistency was further assessed separately for each domain using Cronbach's α. Construct validity was evaluated using confirmatory factor analysis (CFA), and discriminant validity among the three latent constructs was assessed using the HTMT ratio and the Fornell–Larcker criterion.

The final questionnaire, provided in the Supplementary Questionnaire, comprised general characteristics and three KAP domains. General information included sex, age, residence, education, marital status, income type, monthly household income per capita, time since prediabetes diagnosis, hypertension, height, weight, receipt of nutrition-therapy education, and sources of nutrition-therapy information. Body mass index (BMI) was calculated as weight in kilograms divided by height in meters squared and was further categorized as < 24.0, 24.0–27.9, or ≥28.0 kg/m^2^. The perceived knowledge domain contained 10 items rated as “very familiar” (2 points), “heard of it” (1 point), or “do not know” (0 points), producing a total score of 0–20. Knowledge was operationalized as perceived knowledge, reflecting self-reported familiarity rather than objectively assessed accuracy. The attitude domain contained eight 5-point Likert items ranging from “strongly disagree” (1 point) to “strongly agree” (5 points), producing a total score of 8–40. The practice domain contained seven items ranging from “never” (1 point) to “always” (5 points), producing a total score of 7–35. Higher scores indicated greater perceived knowledge, more positive attitudes, and better practices. The three domains were scored and analyzed separately, and no overall KAP total score was calculated. For descriptive interpretation, each KAP domain was classified using modified Bloom cut-offs based on the percentage of the maximum possible score (15, 16). Scores < 60% were categorized as low, 60%−79% as moderate, and > = 80% as high. This corresponded to scores of 0–11, 12–15, and 16–20 for perceived knowledge; 8–23, 24–31, and 32–40 for attitude; and 7–20, 21–27, and 28–35 for practice.

### Questionnaire distribution and quality control

Participants were recruited using convenience sampling. The electronic questionnaire was developed and administered through Wenjuanxing (https://www.wjx.cn/), a widely used online survey platform in China, and was distributed via a QR code or web link. For face-to-face recruitment, trained investigators approached potentially eligible patients in outpatient clinics and inpatient wards and invited them to scan the QR code using their mobile devices. The questionnaire link was also distributed through online patient groups for individuals with prediabetes, allowing eligible participants to complete the survey remotely. Before both onsite and online participation, individuals were informed of the study purpose, the anonymous and voluntary nature of participation, confidentiality protections, and their right to withdraw at any time without consequences. When participants required assistance, investigators provided only procedural or technical support without explaining individual items, suggesting responses, or otherwise influencing questionnaire completion. All analytical items were configured as mandatory; therefore, no missing responses were present in the final dataset.

All submitted questionnaires underwent further quality assessment and were excluded if: (1) the completion time was less than 60 seconds; (2) age, height, weight, or other demographic information contained clearly implausible values, such as a reported age of 180 years; (3) responses showing evident mechanical or invariant response patterns, such as selecting the same response option across all questionnaire items; or (4) the common-knowledge attention-check item was answered incorrectly, indicating that the questionnaire may not have been completed attentively. Only questionnaires that satisfied all predefined quality-control criteria were included in the final analysis.

### Sample-size

The sample size was calculated using the formula for cross-sectional studies (17): *n* = *Z*^2^*p*(1–*p*)/*e*^2^. In this formula, n represents the required sample size, Z represents the standard normal deviation corresponding to a 95% confidence level (*Z* = 1.96), p represents the expected proportion, and e represents the allowable margin of error (set at 5%). Because no previous study had specifically evaluated nutrition therapy-related KAP levels among individuals with prediabetes, a conservative estimate of *p* = 0.50 was adopted to maximize the required sample size. Accordingly, the minimum estimated sample size was 384 participants. Considering a potential 10% rate of invalid questionnaires or non-response, the target sample size was increased to 427 participants.

### Statistical analysis

All statistical analyses were performed using R version 4.4.3. CFA and SEM were conducted using the lavaan package with WLSMV estimation. Categorical variables are presented as numbers and percentages, and KAP scores as mean ± standard deviation. Wilcoxon–Mann–Whitney and Kruskal–Wallis tests assessed unadjusted rank-distribution differences between two or more groups, respectively. Spearman correlations assessed relationships between domain scores. Univariable and multivariable linear regression examined demographic and clinical correlates of observed scores. The regression candidate set comprised sex, age, education, income type, monthly household income per capita, time since diagnosis, hypertension, BMI, and nutrition-therapy education. Residence and marital status were omitted because the distribution of participants across subgroups was imbalanced, raising concerns about the precision of subgroup-specific estimates. Candidate factors with an overall univariable regression *P* < 0.05 were entered into multivariable models. Perceived knowledge was additionally included in the attitude model, and perceived knowledge and attitude in the practice model. Regression coefficients are reported as unstandardized B. Variance inflation factors and tolerance values were examined for multicollinearity, and residual variance diagnostics were assessed. HC3 robust intervals retaining the original adjustment variables were used for supplementary sensitivity analyses.

SEM addressed relationships among latent KAP constructs, whereas regression addressed correlates of observed scores. All 25 indicators were treated as ordered; CFA and SEM used WLSMV estimation with delta parameterization and the same six prespecified residual covariances. Model fit was evaluated using scaled chi-square (χ^2^), the comparative fit index (CFI), Tucker–Lewis index (TLI), root mean square error of approximation (RMSEA), their robust counterparts where applicable, and the standardized root mean square residual (SRMR). Direct-path intervals used robust delta-method standard errors. Indirect and total association intervals used 100,000 Monte Carlo draws from the joint asymptotic distribution of the parameter estimates. Domain-specific Cronbach's α with 2,000-resample bootstrap intervals were calculated. Discriminant validity was assessed using polychoric HTMT and the Fornell–Larcker criterion. All tests were two-sided, and P < 0.05 was considered statistically significant.

## Results

### Reliability and validity of the questionnaire

In the main sample, Cronbach's α was 0.946, 0.872, and 0.920 for perceived knowledge, attitude, and practice, respectively (Table S1). HTMT values ranged from 0.568 to 0.737, all below the 0.85 threshold. The Fornell–Larcker criterion was satisfied for the perceived knowledge–practice and attitude–practice pairs. For perceived knowledge–attitude, the latent correlation (0.744) slightly exceeded the square root of attitude AVE (0.729), although it remained below that of perceived knowledge (0.864). Overall, discriminant validity was generally supported, with some overlap between perceived knowledge and attitude (Table S2). CFA was performed using all 449 participants in the main survey to assess the measurement model used in SEM. The six residual covariances were specified before analysis on substantive grounds (Table S3). The CFA yielded scaled CFI = 0.985, TLI = 0.983, RMSEA = 0.060, and SRMR = 0.041, whereas the corresponding robust CFI, TLI, and RMSEA were 0.902, 0.889, and 0.096, respectively (Table S4). Standardized factor loadings ranged from 0.528 to 0.943, and all were statistically significant (Table S5).

### Participant characteristics

A total of 510 questionnaires were initially collected. Of these, 61 were excluded: four because the respondents were younger than 18 years, 25 because of incorrect responses to the attention-check item, 13 because the completion time was less than 60 s, eight because of implausible or abnormal data, and 11 because of evident mechanical or repetitive response patterns. Consequently, 449 valid questionnaires were included in the final analysis, corresponding to a valid rate of 88.0%. The sample comprised 257 women (57.2%) and 331 participants (73.7%) were aged < 60 years. Most participants lived in urban areas (432, 96.2%) and were married (415, 92.4%). Only 95 participants (21.2%) had received prior nutrition-therapy education. The most frequently reported sources of nutrition-therapy information were healthcare professionals (410, 91.3%), followed by family or friends (278, 61.9%), news or television (235, 52.3%), and social media (128, 28.5%) (Table 1).

**Table 1:** Characteristics and KAP scores of the participants.

Variable	*N* (%)	Perceived knowledge, mean ±SD	*P*-value	Attitude, mean ±SD	*P*-value	Practice, mean ±SD	*P*-value
**Total score**	449 (100.0%)	12.03 ± 5.51		27.21 ± 4.79		22.71 ± 5.60	
**Sex**			0.456		0.921		0.418
Male	192 (42.8%)	12.27 ± 5.44		27.21 ± 4.69		23.16 ± 5.11	
Female	257 (57.2%)	11.85 ± 5.56		27.21 ± 4.87		22.38 ± 5.92	
**Age**			0.843		0.165		0.010
< 60 years	331 (73.7%)	12.02 ± 5.71		27.34 ± 4.85		22.27 ± 5.68	
≥60 years	118 (26.3%)	12.06 ± 4.94		26.85 ± 4.61		23.97 ± 5.16	
**Residence**			0.076		0.043		0.084
Urban	432 (96.2%)	11.94 ± 5.51		27.13 ± 4.79		22.77 ± 5.62	
Rural	17 (3.8%)	14.18 ± 5.32		29.12 ± 4.40		21.24 ± 4.82	
**Educational level**			0.038		0.280		0.493
Junior high school or below	213 (47.4%)	12.52 ± 5.20		27.25 ± 4.81		23.11 ± 5.23	
High school/technical secondary school	160 (35.6%)	12.11 ± 5.70		27.47 ± 4.69		22.46 ± 5.96	
Junior college or above	76 (16.9%)	10.49 ± 5.74		26.54 ± 4.93		22.13 ± 5.80	
**Marital status**			0.779		0.087		0.006
Married	415 (92.4%)	12.07 ± 5.51		27.30 ± 4.83		22.93 ± 5.47	
Other	34 (7.6%)	11.59 ± 5.59		26.03 ± 4.14		20.03 ± 6.50	
**Income type**			< 0.001		0.017		< 0.001
No stable income	166 (37.0%)	10.81 ± 5.61		26.51 ± 4.57		20.93 ± 5.81	
Stable income	283 (63.0%)	12.74 ± 5.33		27.61 ± 4.88		23.76 ± 5.20	
**Monthly household income per capita**			0.046		0.881		0.934
< 2,000 CNY	81 (18.0%)	13.32 ± 5.09		27.58 ± 4.42		23.06 ± 4.79	
2,000–4,999 CNY	223 (49.7%)	11.96 ± 5.67		27.07 ± 4.96		22.56 ± 5.74	
≥5,000 CNY	145 (32.3%)	11.41 ± 5.40		27.21 ± 4.75		22.77 ± 5.81	
**Time since prediabetes diagnosis**			< 0.001		< 0.001		0.067
< 3 months	128 (28.5%)	10.50 ± 5.12		25.95 ± 4.53		21.84 ± 6.69	
3–6 months	87 (19.4%)	13.33 ± 5.84		28.24 ± 4.39		23.86 ± 4.48	
>6 months−2 years	111 (24.7%)	12.60 ± 6.23		27.86 ± 5.31		23.10 ± 5.87	
>2 years	123 (27.4%)	12.18 ± 4.59		27.20 ± 4.60		22.47 ± 4.61	
**Hypertension**			0.449		0.720		0.073
No	130 (29.0%)	11.48 ± 6.07		27.12 ± 5.12		21.60 ± 6.70	
Yes	319 (71.0%)	12.25 ± 5.26		27.24 ± 4.66		23.17 ± 5.02	
**Body mass index**			0.928		0.865		0.646
< 24.0 kg/m^2^	222 (49.4%)	12.10 ± 5.45		27.19 ± 5.11		22.63 ± 5.56	
24.0–27.9 kg/m^2^	174 (38.8%)	11.98 ± 5.38		27.13 ± 4.54		22.70 ± 5.49	
≥28.0 kg/m^2^	53 (11.8%)	11.91 ± 6.22		27.53 ± 4.27		23.13 ± 6.17	
**Nutrition-therapy education**			< 0.001		< 0.001		0.483
No	354 (78.8%)	11.03 ± 5.27		26.77 ± 4.89		22.70 ± 5.76	
Yes	95 (21.2%)	15.75 ± 4.76		28.85 ± 4.00		22.77 ± 4.96	

Data are presented as n (%) for categorical variables and mean ± SD for perceived knowledge, attitude, and practice scores. Between-group comparisons were performed using the Wilcoxon–Mann–Whitney U-test for two-group variables and the Kruskal–Wallis test for variables with more than two groups. These P-values compare rank distributions, not mean differences.

The mean scores were 12.03 ± 5.51 for perceived knowledge (possible range: 0–20), 27.21 ± 4.79 for attitude (possible range: 8–40), and 22.71 ± 5.60 for practice (possible range: 7–35). According to modified Bloom cut-offs, the majority of participants held low perceived knowledge (239, 53.2%), while moderate attitudes (291, 64.8%) and moderate practices (250, 55.7%) were the predominant categories (Table S6). Perceived knowledge scores differed across educational level, income type, monthly household income, time since diagnosis, and nutrition-therapy education groups (all *P* < 0.05). Attitude scores differed by residence, income type, time since diagnosis, and nutrition-therapy education (all *P* < 0.05). Practice scores differed by age, marital status, and income type (all *P* < 0.05) (Table 1).

### Item-level response patterns

The perceived knowledge item with the highest observed proportion of “do not know” responses concerned eating vegetables first, protein foods second, and staple foods last to reduce postprandial glycemic responses (K10; 87, 19.4%). Relatively high proportions of “do not know” responses were also observed for the ability of nutrition therapy and lifestyle intervention to delay or prevent diabetes (K2; 81, 18.0%), the effect of high-glycemic-index foods on postprandial glucose (K8; 73, 16.3%), and the role of dietary fiber in slowing glucose elevation and improving insulin sensitivity (K3; 72, 16.0%). The highest observed proportion of “very familiar” responses was observed for the adverse effects of excessive refined sugar and sugar-sweetened beverage intake (K7; 207, 46.1%) (Table 2).

**Table 2:** Distribution of perceived knowledge responses.

Items, *n* (%)	Very familiar	Heard of it	Do not know
1. Prediabetes is a condition between normal blood glucose and diabetes.	109 (24.3%)	280 (62.4%)	60 (13.4%)
2. Nutrition therapy and lifestyle intervention during prediabetes can delay or prevent diabetes.	149 (33.2%)	219 (48.8%)	81 (18.0%)
3. Dietary fiber from whole grains, legumes, and vegetables can slow glucose elevation and improve insulin sensitivity.	109 (24.3%)	268 (59.7%)	72 (16.0%)
4. People with prediabetes need to control total energy intake.	166 (37.0%)	226 (50.3%)	57 (12.7%)
5. Regular meals with consistent timing and portions are more beneficial for glycemic control.	169 (37.6%)	230 (51.2%)	50 (11.1%)
6. People with prediabetes who are overweight or obese need to prioritize weight management.	174 (38.8%)	204 (45.4%)	71 (15.8%)
7. Excessive intake of refined sugar and sugar-sweetened beverages is unfavorable for prediabetes control.	207 (46.1%)	185 (41.2%)	57 (12.7%)
8. High-glycemic-index foods such as white bread and sugar-sweetened beverages rapidly increase postprandial glucose.	162 (36.1%)	214 (47.7%)	73 (16.3%)
9. People with prediabetes should combine dietary intervention with physical activity.	190 (42.3%)	204 (45.4%)	55 (12.2%)
10. Eating vegetables first, protein foods second, and staple foods last can reduce postprandial glucose responses.	139 (31.0%)	223 (49.7%)	87 (19.4%)

Data are presented descriptively as n (%); no inferential statistical tests were performed.

For the attitude dimension, the highest observed combined proportions of “disagree” and “strongly disagree” were observed for Item A1, initiating intervention early during the prediabetes stage (61, 13.6%); and Item A3, confidence in adhering to a healthy eating plan (57, 12.7%). Item A8, the benefit of combining exercise and diet, had a disagreement proportion (56, 12.5%) and 208 neutral responses (46.3%). Overall, relatively high proportions of favorable responses were observed for items concerning the perceived importance and effectiveness of nutrition therapy and the value of dietary control before diabetes develops (A2, A4, and A7) (Table 3).

**Table 3:** Distribution of attitude dimension responses.

Items, *n* (%)	Strongly agree	Agree	Neutral	Disagree	Strongly disagree
1. I believe that intervention should begin early during the prediabetes stage.	14 (3.1%)	162 (36.1%)	212 (47.2%)	57 (12.7%)	4 (0.9%)
2. I believe that nutrition therapy is important for delaying or preventing diabetes.	53 (11.8%)	187 (41.6%)	168 (37.4%)	35 (7.8%)	6 (1.3%)
3. I am confident that I can adhere to a healthy eating plan.	53 (11.8%)	177 (39.4%)	162 (36.1%)	52 (11.6%)	5 (1.1%)
4. I believe that nutrition therapy can effectively control or reverse prediabetes and prevent progression to diabetes.	38 (8.5%)	156 (34.7%)	202 (45.0%)	51 (11.4%)	2 (0.4%)
5. I am willing to adjust my diet under the guidance of a physician or dietitian.	23 (5.1%)	178 (39.6%)	194 (43.2%)	49 (10.9%)	5 (1.1%)
6. Compared with medication, I prefer to manage blood glucose through diet and lifestyle changes.	25 (5.6%)	159 (35.4%)	210 (46.8%)	49 (10.9%)	6 (1.3%)
7. I believe that dietary control during prediabetes is more meaningful than waiting to treat diabetes after it develops.	60 (13.4%)	156 (34.7%)	179 (39.9%)	44 (9.8%)	10 (2.2%)
8. I believe that combining exercise with diet produces better results.	20 (4.5%)	165 (36.7%)	208 (46.3%)	53 (11.8%)	3 (0.7%)

Data are presented descriptively as n (%); no inferential statistical tests were performed.

For the practice dimension, the highest observed combined proportions of “rarely” and “never” were observed for Item P6, following the recommended meal sequence of vegetables first, meat or eggs second, and staple foods last (101, 22.5%); Item P5, checking nutrition information on food packaging (92, 20.5%); and Item P4, controlling staple-food portions while combining refined and whole-grain foods (89, 19.8%). Overall, relatively high proportions of favorable practice responses were observed for adjusting daily food intake, monitoring body weight, and actively seeking dietary information (P3, P7, and P1) (Table 4).

**Table 4:** Distribution of practice dimension responses.

Items, *n* (%)	Always	Often	Sometimes	Rarely	Never
1. I actively seek dietary information suitable for prediabetes.	19 (4.2%)	171 (38.1%)	187 (41.6%)	51 (11.4%)	21 (4.7%)
2. I measure my blood glucose regularly.	16 (3.6%)	175 (39.0%)	170 (37.9%)	60 (13.4%)	28 (6.2%)
3. I control my daily food intake according to my own condition.	39 (8.7%)	189 (42.1%)	144 (32.1%)	49 (10.9%)	28 (6.2%)
4. I consciously control staple-food portions and combine refined and whole-grain staple foods.	37 (8.2%)	160 (35.6%)	163 (36.3%)	61 (13.6%)	28 (6.2%)
5. I check nutrition information on food packaging.	25 (5.6%)	161 (35.9%)	171 (38.1%)	55 (12.2%)	37 (8.2%)
6. I eat vegetables first, meat or eggs second, and staple foods last.	20 (4.5%)	149 (33.2%)	179 (39.9%)	66 (14.7%)	35 (7.8%)
7. I record weight changes or pay attention to weight management.	40 (8.9%)	173 (38.5%)	161 (35.9%)	46 (10.2%)	29 (6.5%)

Data are presented descriptively as n (%); no inferential statistical tests were performed.

### Factors associated with KAP scores

In multivariable linear regression analysis, perceived knowledge scores were significantly higher among participants with a stable income (*B* = 1.880, 95% CI: 0.839–2.921, *P* < 0.001), those diagnosed 3–6 months previously (*B* = 2.705, 95% CI: 1.338–4.072), >6 months−2 years previously (*B* = 1.794, 95% CI: 0.467–3.122), or >2 years previously (*B* = 1.413, 95% CI: 0.113–2.712), and those who had received prior nutrition-therapy education (*B* = 4.774, 95% CI: 3.633–5.915, *P* < 0.001), whereas participants with a monthly household income per capita of 2,000–4,999 yuan had lower perceived knowledge scores (*B* = −1.355, 95% CI: −2.706–−0.003, *P* = 0.049) (Table S7).

The multivariable attitude model included income type, time since diagnosis, prior nutrition-therapy education, and perceived knowledge. Only perceived knowledge was significantly associated with attitude score (*B* = 0.578, 95% CI: 0.510–0.646, *P* < 0.001) (Table S8).

Practice scores were significantly higher among participants aged ≥60 years (*B* = 1.457, 95% CI: 0.529–2.385, *P* = 0.002) and those with a stable income (*B* = 1.603, 95% CI: 0.762–2.445, *P* < 0.001), but lower among participants without hypertension (*B* = −1.004, 95% CI: −1.895–−0.113, *P* = 0.027); higher perceived knowledge scores (*B* = 0.258, 95% CI: 0.162–0.354, *P* < 0.001) and attitude scores (*B* = 0.464, 95% CI: 0.355–0.574, *P* < 0.001) were independently associated with better practices (Table S9). The original regression coefficients retained their directions and nominal significance patterns with HC3 standard errors (Tables S10, S11). No substantial multicollinearity was detected in the practice regression model; the VIFs for perceived knowledge and attitude were 1.787 and 1.757, respectively.

### Correlations among perceived knowledge, attitudes, and practices

Spearman correlation analysis showed that perceived knowledge scores were positively correlated with attitude scores (*r* = 0.617, *P* < 0.001) and practice scores (*r* = 0.427, *P* < 0.001). Attitude scores were also positively correlated with practice scores (*r* = 0.494, *P* < 0.001) (Table S12).

### Structural equation model

The WLSMV SEM yielded a scaled χ^2^ of 699.513 (df = 266), scaled CFI = 0.985, TLI = 0.983, and RMSEA = 0.060 (90% CI: 0.055–0.066), with SRMR = 0.041. Robust CFI = 0.902, TLI = 0.889, and RMSEA = 0.096 (90% CI: 0.089–0.103) indicated that overall fit was not uniformly satisfactory (Table S13). Perceived knowledge was positively associated with attitude (β = 0.744, 95% CI: 0.698–0.790), supporting H1, and with practice (β = 0.226, 95% CI: 0.107–0.345), supporting H2. Attitude was positively associated with practice (β = 0.469, 95% CI: 0.351–0.587), supporting H3 (all *P* < 0.001). The indirect association of perceived knowledge with practice through attitude was 0.349 (Monte Carlo 95% CI: 0.257–0.444), supporting H4 (Figure 1, Table 5, and Table S14).

**Figure 1:**
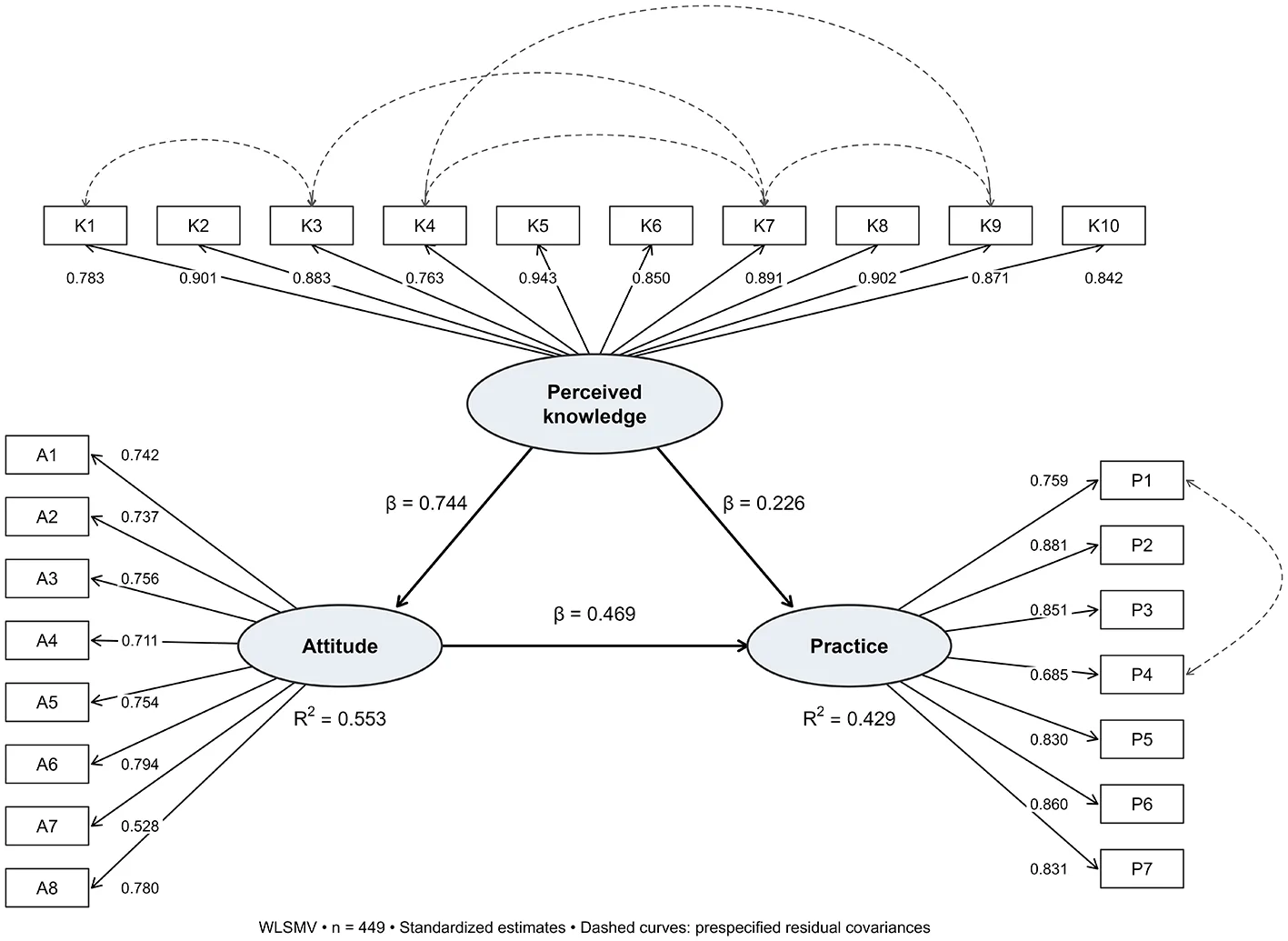
Structural equation model of perceived knowledge, attitude, and practice. Ellipses denote latent constructs and rectangles denote ordered questionnaire items. Single-headed arrows show standardized structural coefficients (β) or standardized factor loadings. Dashed double-headed curves denote the six prespecified residual covariances, detailed in Table S3. Residual nodes and fixed unit paths are omitted for readability. *R*^2^ values are shown for attitude and practice. Arrows indicate the hypothesized model ordering, not established causal direction.

**Table 5:** Structural associations in the WLSMV model.

Path	*B*	SE (*B*)	95% CI (*B*)	P (*B*)	β	SE (β)	95% CI (β)	P (β)
Perceived knowledge → attitude	0.742	0.035	0.673–0.810	< 0.001	0.744	0.023	0.698–0.790	< 0.001
Perceived knowledge → practice	0.219	0.060	0.102–0.336	< 0.001	0.226	0.061	0.107–0.345	< 0.001
Attitude → practice	0.456	0.061	0.337–0.575	< 0.001	0.469	0.060	0.351–0.587	< 0.001

B, unstandardized coefficient; β, standardized coefficient; SE, robust standard error; CI, confidence interval. Direct-path intervals use the robust delta method. Attitude R^2^ = 0.553; practice R^2^ = 0.429. All estimates refer to the same model with six prespecified residual covariances.

## Discussion

In this study, the majority of participants with prediabetes reported low nutrition-therapy perceived knowledge, while attitudes and practices were predominantly moderate. The structural model was consistent with the prespecified KAP pathway: perceived knowledge was linked to practices directly and indirectly through attitudes. Thus, the contribution of this study lies not only in characterizing nutrition therapy-related KAP in an understudied prediabetes population, but also in examining the interrelationships among these constructs within an item-level latent-variable framework. Together, these findings provide a more integrated understanding of nutrition therapy-related KAP in adults with prediabetes and may help inform the development of future nutrition-education strategies.

Our results broadly accord with previous nutrition-related KAP research. In a national survey of 6,441 patients with diabetes from urban China, more than half had poor medical nutrition therapy KAP, and exposure to nutrition education was associated with better scores (13). A systematic review of dietary practices in people with type 2 diabetes similarly identified nutrition education, knowledge, attitudes, and socioeconomic resources as correlates of better dietary practice (14). Direct numerical comparisons are inappropriate because populations, disease stage, healthcare exposure, instruments, scoring systems, and thresholds differed. Individuals with established diabetes may also have longer exposure to treatment and nutrition education than those with prediabetes. Despite these differences, the recurring pattern across studies supports the practical importance of education and of addressing barriers that prevent knowledge from being applied.

More than half of the participants were classified as having low nutrition-therapy perceived knowledge, indicating limited self-reported familiarity with dietary management during prediabetes. Relatively higher proportions of “do not know” responses were observed for items concerning meal sequence (K10), the preventive value of nutrition and lifestyle intervention (K2), high-glycemic-index foods (K8), and dietary fiber (K3). This pattern is consistent with a South Indian study in which 90% of individuals with prediabetes had low baseline perceived knowledge and with a large Chinese survey reporting low medical nutrition therapy perceived knowledge among patients with diabetes (13, 18). The relatively limited perceived knowledge observed in our study may be related to differences in previous exposure to nutrition-therapy education, although the cross-sectional design does not allow this relationship to be interpreted causally. The observed results should not be interpreted as being limited to these individual topics, because the questionnaire assessed only selected specific aspects of nutrition therapy and did not capture the full range of dietary perceived knowledge required for prediabetes management. These responses identify perceived familiarity gaps but do not establish whether participants could answer objective knowledge questions correctly. Therefore, nutrition education may consider adopting a comprehensive approach that covers the principles, rationale, and practical application of dietary management.

Attitudes toward nutrition therapy were predominantly moderate. Relatively higher proportions of unfavorable or neutral responses were observed for items concerning the need for early intervention (A1), confidence in maintaining a healthy eating plan (A3), and belief in the additional benefit of combining diet with exercise (A8). Similar findings have been reported in a recent study in which approximately half of participants demonstrated neutral attitudes toward prediabetes, while a systematic review found that generally favorable views of diabetes prevention often coexisted with limited motivation, unclear goals, and barriers to behavioral change (19, 20). Previous studies have identified barriers such as limited motivation, uncertainty about feasibility, food preferences, low self-efficacy, and difficulties sustaining dietary changes (19, 21). These factors were not directly assessed in the present study and are discussed only as potential contextual explanations for the observed response patterns. Future educational programs may incorporate risk communication, collaborative goal setting, and confidence-building rather than relying solely on the transmission of nutritional information.

Nutrition-related practices were also predominantly moderate, with relatively higher proportions of “rarely” or “never” responses observed for adherence to the recommended meal sequence (P6), checking nutrition labels (P5), and controlling staple-food portions while combining refined and whole-grain foods (P4). These findings indicate that general dietary intentions may not readily translate into specific and sustained self-management behaviors. Previous evidence similarly shows that positive knowledge and attitudes toward prediabetes are frequently accompanied by inadequate preventive practices; one Saudi study reported poor diet and lifestyle practices in 84% of participants, while a prediabetes education study demonstrated substantial improvements in practice after counseling (18, 21). These international findings provide context rather than direct benchmarks, given differences in dietary culture, health-care delivery, sampling, and measurement. Published literature has also identified practical barriers, including limited nutritional skills, difficulty interpreting food labels, established eating habits, and insufficient follow-up (19, 21). These barriers were not directly measured in the present study. Future nutrition support may include repeated, skills-based guidance, including meal planning, portion control, food-label interpretation, culturally appropriate substitutions, and self-monitoring tools to facilitate the application of recommendations in daily life.

The results of multivariable linear regression highlighted that prior nutrition-therapy education showed the largest independent association with perceived knowledge in our study. Participants who had received prior nutrition-therapy education scored nearly five points higher on a 20-point scale. Because the questionnaire did not record the source, format, duration, or content of that education, this association should not be interpreted as evidence of the effect of any specific educational provider or intervention. Current guidelines recommend individualized nutrition counseling and continuing support from qualified healthcare professionals (7, 8). Reverse causation and selection also remain plausible, as people who are more health conscious, have greater access to care, or have lived longer with prediabetes may be more likely to seek education. Accordingly, prior education should be regarded as a potentially important intervention target rather than evidence of a causal effect on perceived knowledge.

Apart from prior nutrition-therapy education, several demographic and clinical associations also warrant cautious interpretation. Older participants reported better practices, consistent with previous evidence showing that older adults with diabetes generally demonstrate better dietary adherence, glucose monitoring, and overall self-management than younger adults (22, 23). Time since prediabetes diagnosis, however, was not independently associated with practice, indicating that the age association should not be attributed simply to longer disease exposure. The mechanisms underlying this association were not directly assessed. Stable income was associated with both perceived knowledge and practices. Previous research has linked higher socioeconomic status with greater nutrition knowledge, healthier dietary patterns, and better diabetes self-management, whereas socioeconomically disadvantaged individuals with prediabetes were less likely to access dietetic services (24). Income stability and income amount, however, represent distinct socioeconomic indicators and should not be interpreted interchangeably. Participants with hypertension also reported better practices. Although greater exposure to dietary or lifestyle counseling is one possible explanation, the duration of hypertension and prior hypertension-related counseling were not assessed. This interpretation therefore remains hypothetical.

Perceived knowledge and attitudes were independently associated with practices in both regression and SEM analyses. The standardized path from attitudes to practices was larger than the direct path from perceived knowledge to practices, while perceived knowledge retained a direct association and a substantial indirect association through attitudes. This pattern is conceptually consistent with KAP models in which perceived knowledge provides a cognitive basis for behavior, while attitudes reflect acceptance and motivation (11, 25). The findings describe associations among perceived familiarity, attitudes, and practices, rather than demonstrating a behavioral mechanism. Our SEM tested whether the data were consistent with the prespecified theoretical ordering, but it does not establish that perceived knowledge preceded attitudes or that attitudes mediated a causal effect in this cross-sectional study. Similar relationships among knowledge, attitudes, and practices have been reported in other chronic-disease populations. In older adults with sarcopenia, SEM demonstrated positive paths from knowledge to attitude and practice and from attitude to practice, together with an indirect knowledge–practice pathway through attitude (26). Comparable patterns have also been observed in hemodialysis patients (27) and in patients with diabetes mellitus and hyperuricemia (28), in whom knowledge and attitudes were positively related to practice and attitude contributed to the indirect association between knowledge and practice. These findings suggest that the relationships observed in our study may reflect a broader KAP-related behavioral pattern rather than one specific to prediabetes, although differences in disease context, populations, and measurement instruments preclude direct quantitative comparison.

The findings have several practical implications. First, clinics could briefly assess nutrition-therapy perceived knowledge and practices when prediabetes is diagnosed. Second, future nutrition-education programs may consider addressing topics represented by items with relatively high proportions of unfavorable responses, including early preventive value, dietary fiber, glycemic index, and meal sequence, together with established components of prediabetes nutrition management such as energy balance and weight management. Third, counseling could use familiar food packages to practice comparing serving sizes, carbohydrate, and fiber information (P5), and household bowls or plates to demonstrate staple-food portions and whole-grain substitutions (P4), consistent with individualized nutrition guidance (6–8). For meal sequencing (P6), healthcare professionals could demonstrate eating vegetables and protein before carbohydrate foods. These activities are proposed educational strategies rather than interventions tested in the present survey. Fourth, counseling may also consider addressing attitudes and self-efficacy through collaborative goals and feasible stepwise changes. Although healthcare professionals were the most common information source, more than half of participants also used family, news, or television sources and almost one-third used social media. Given this diversity of information sources, nutrition education may also include guidance on evaluating potentially inconsistent information and identifying reliable materials. Because the questionnaire assessed selected rather than exhaustive aspects of prediabetes-related nutrition knowledge and practices, these item-specific findings should not be regarded as a complete set of educational priorities. Future educational programs and research should also consider broader guideline-recommended topics that were not specifically assessed in the present questionnaire, including sodium and saturated fat intake, overall dietary quality, and other relevant components of cardiometabolic risk management. Further studies incorporating these domains are needed to determine whether additional knowledge or practice gaps exist.

Several limitations should be acknowledged. First, the cross-sectional design precludes conclusions about temporality, causality, or mediation. Because the SEM was based on cross-sectional data, the directional paths should be interpreted cautiously and should not be considered evidence of causal relationships. Second, recruitment from a single center using convenience sampling may have introduced selection bias, and 96.2% of participants were urban residents, limiting generalizability to rural and other populations. Third, practices were self-reported and may have been affected by recall and social-desirability bias. No dietary records, objective activity measures, or biomarkers were used to verify behavior. Fourth, perceived knowledge measured familiarity, not objective accuracy. Fifth, expert review and pilot feedback provided qualitative content and comprehensibility assessment, but no quantitative content validity index or external criterion validation was available. CFA and SEM used the same sample and do not constitute independent replication. Discriminant validity was generally supported by HTMT and the Fornell–Larcker criterion, although the perceived knowledge–attitude pair showed some overlap and did not fully satisfy the Fornell–Larcker criterion. The questionnaire therefore requires further independent validation, including assessment of test–retest reliability. Sixth, potentially important variables such as glycated hemoglobin, fasting glucose, medication use, detailed dietary intake, physical activity, family history of diabetes, healthcare exposure, and comorbidities were not collected, leaving residual confounding. Also, the source, format, duration, and content of previous nutrition-therapy education were not assessed, limiting interpretation of this variable. Seventh, regression diagnostics indicated heteroscedasticity, although HC3 sensitivity analyses yielded consistent results. Finally, although the questionnaire covered several guideline-based nutrition topics, it was not designed to exhaustively assess all aspects of prediabetes-related nutrition knowledge, and some clinically relevant topics, such as sodium and saturated fat intake, were not specifically included. Future research should use multicenter probability or stratified sampling with greater rural representation, externally validate the questionnaire, and combine self-reported KAP with dietary intake, physical activity, body weight, and glycemic outcomes. Longitudinal studies are needed to establish temporal ordering.

## Conclusions

Participants in this study reported limited nutrition-therapy perceived knowledge, while attitudes and practices were mainly moderate. Prior nutrition-therapy education was strongly associated with perceived knowledge, and perceived knowledge and attitudes were independently associated with practices. Within the specified structural model, perceived knowledge was associated with practices directly and indirectly through attitudes; these associations require cautious interpretation given the measurement and cross-sectional limitations. These findings suggest that future nutrition-education interventions may consider addressing perceived knowledge, attitudes, and practical dietary behaviors, although their effectiveness requires prospective evaluation.

## Data Availability

The raw data supporting the conclusions of this article will be made available by the authors, without undue reservation.
