# Supplementary material for: Perceived knowledge, attitudes, and practices regarding nutrition therapy among people with prediabetes: a structural equation model analysis
Source: Front Nutr. 2026 Oct 29;13:1952271. doi: 10.3389/fnut.2026.1952271 (PMC13754649; doi:10.3389/fnut.2026.1952271)
Supplement: Supplementary file 1 [file Table_1.docx]

**Table S1. Reliability in the main sample**

| Domain | N | Items | α | 95% CI for α |
| --- | --- | --- | --- | --- |
| Perceived knowledge | 449 | 10 | 0.946 | 0.935 to 0.955 |
| Attitude | 449 | 8 | 0.872 | 0.843 to 0.895 |
| Practice | 449 | 7 | 0.920 | 0.901 to 0.935 |

α intervals use 2,000 participant-level bootstrap resamples.

**Table S2. Discriminant validity**

| Construct pair | Latent r (95% CI) | √AVE, first / second | Polychoric HTMT | Fornell–Larcker |
| --- | --- | --- | --- | --- |
| Perceived knowledge / Attitude | 0.744 (0.698 to 0.790) | 0.864 / 0.729 | 0.737 | Not fully met |
| Perceived knowledge / Practice | 0.575 (0.518 to 0.631) | 0.864 / 0.816 | 0.568 | Met |
| Attitude / Practice | 0.637 (0.584 to 0.690) | 0.729 / 0.816 | 0.638 | Met |

AVE: perceived knowledge 0.747, attitude 0.532, practice 0.666.

**Table S3. Prespecified residual covariances and content rationale**

| Items | Content rationale | Covariance | SE | 95% CI | P | Std. residual correlation |
| --- | --- | --- | --- | --- | --- | --- |
| P1–P4 | Active dietary-information seeking and implementation of staple-food planning may share a specific dietary self-management component. | 0.182 | 0.025 | 0.133 to 0.231 | <0.001 | 0.384 |
| K7–K9 | Both address actionable lifestyle-management advice; the shared content is broader than a direct wording overlap. | 0.100 | 0.019 | 0.063 to 0.137 | <0.001 | 0.449 |
| K4–K9 | Total energy-intake control and combined dietary/physical-activity intervention share energy-balance management content. | 0.185 | 0.023 | 0.141 to 0.229 | <0.001 | 0.582 |
| K4–K7 | General energy-intake restriction and limiting refined sugar/sweetened drinks share intake-control content. | 0.135 | 0.022 | 0.092 to 0.178 | <0.001 | 0.460 |
| K1–K3 | Both concern glycemic status or regulation; one is a disease definition and the other a nutritional mechanism, so the content connection is indirect. | 0.170 | 0.024 | 0.123 to 0.216 | <0.001 | 0.581 |
| K3–K7 | Both concern carbohydrate sources or quality in glycemic management, contrasting fiber-rich foods with refined sugar sources. | -0.060 | 0.015 | -0.089 to -0.031 | <0.001 | -0.282 |

**Table S4. CFA fit indices**

| Index | Scaled | Robust |
| --- | --- | --- |
| Scaled χ² / df | 699.513 / 266 | — |
| CFI | 0.985 | 0.902 |
| TLI | 0.983 | 0.889 |
| RMSEA (90% CI) | 0.060 (0.055 to 0.066) | 0.096 (0.089 to 0.103) |
| SRMR | 0.041 | — |

N=449. All indicators were ordered. WLSMV estimation with delta parameterization and six prespecified residual covariances. Scaled and robust fit indices are distinct calculations and are therefore reported separately. SRMR is reported as a single value.

Abbreviations: CFA, confirmatory factor analysis; χ², chi-square; df, degrees of freedom; CFI, comparative fit index; TLI, Tucker–Lewis index; RMSEA, root mean square error of approximation; CI, confidence interval; SRMR, standardized root mean square residual; WLSMV, weighted least squares mean- and variance-adjusted.

**Table S5. CFA factor loadings**

| Construct | Item | B | SE(B) | 95% CI(B) | P(B) | β | SE(β) | 95% CI(β) | P(β) |
| --- | --- | --- | --- | --- | --- | --- | --- | --- | --- |
| Perceived knowledge | K1 | 1.000 | Fixed | — | — | 0.783 | 0.025 | 0.734 to 0.832 | <0.001 |
| Perceived knowledge | K2 | 1.151 | 0.037 | 1.079 to 1.223 | <0.001 | 0.901 | 0.012 | 0.878 to 0.924 | <0.001 |
| Perceived knowledge | K3 | 1.128 | 0.033 | 1.063 to 1.193 | <0.001 | 0.883 | 0.016 | 0.852 to 0.914 | <0.001 |
| Perceived knowledge | K4 | 0.975 | 0.038 | 0.901 to 1.049 | <0.001 | 0.763 | 0.023 | 0.718 to 0.808 | <0.001 |
| Perceived knowledge | K5 | 1.204 | 0.037 | 1.131 to 1.277 | <0.001 | 0.943 | 0.010 | 0.924 to 0.962 | <0.001 |
| Perceived knowledge | K6 | 1.086 | 0.038 | 1.010 to 1.161 | <0.001 | 0.850 | 0.017 | 0.816 to 0.884 | <0.001 |
| Perceived knowledge | K7 | 1.139 | 0.038 | 1.064 to 1.213 | <0.001 | 0.891 | 0.014 | 0.864 to 0.918 | <0.001 |
| Perceived knowledge | K8 | 1.152 | 0.035 | 1.083 to 1.222 | <0.001 | 0.902 | 0.011 | 0.880 to 0.925 | <0.001 |
| Perceived knowledge | K9 | 1.113 | 0.036 | 1.042 to 1.183 | <0.001 | 0.871 | 0.015 | 0.842 to 0.901 | <0.001 |
| Perceived knowledge | K10 | 1.075 | 0.035 | 1.007 to 1.143 | <0.001 | 0.842 | 0.016 | 0.810 to 0.873 | <0.001 |
| Attitude | A8 | 1.000 | Fixed | — | — | 0.780 | 0.020 | 0.742 to 0.819 | <0.001 |
| Attitude | A1 | 0.951 | 0.035 | 0.882 to 1.020 | <0.001 | 0.742 | 0.021 | 0.701 to 0.783 | <0.001 |
| Attitude | A2 | 0.944 | 0.031 | 0.883 to 1.005 | <0.001 | 0.737 | 0.020 | 0.697 to 0.776 | <0.001 |
| Attitude | A3 | 0.968 | 0.033 | 0.903 to 1.034 | <0.001 | 0.756 | 0.022 | 0.712 to 0.799 | <0.001 |
| Attitude | A4 | 0.911 | 0.036 | 0.840 to 0.981 | <0.001 | 0.711 | 0.022 | 0.668 to 0.753 | <0.001 |
| Attitude | A5 | 0.966 | 0.029 | 0.909 to 1.023 | <0.001 | 0.754 | 0.020 | 0.715 to 0.792 | <0.001 |
| Attitude | A6 | 1.017 | 0.031 | 0.957 to 1.077 | <0.001 | 0.794 | 0.019 | 0.757 to 0.831 | <0.001 |
| Attitude | A7 | 0.677 | 0.045 | 0.589 to 0.765 | <0.001 | 0.528 | 0.033 | 0.464 to 0.593 | <0.001 |
| Practice | P1 | 1.000 | Fixed | — | — | 0.759 | 0.022 | 0.717 to 0.801 | <0.001 |
| Practice | P2 | 1.161 | 0.033 | 1.096 to 1.227 | <0.001 | 0.881 | 0.013 | 0.856 to 0.907 | <0.001 |
| Practice | P3 | 1.122 | 0.035 | 1.053 to 1.190 | <0.001 | 0.851 | 0.015 | 0.821 to 0.882 | <0.001 |
| Practice | P4 | 0.902 | 0.030 | 0.844 to 0.960 | <0.001 | 0.685 | 0.025 | 0.636 to 0.733 | <0.001 |
| Practice | P5 | 1.094 | 0.035 | 1.025 to 1.163 | <0.001 | 0.830 | 0.017 | 0.797 to 0.864 | <0.001 |
| Practice | P6 | 1.133 | 0.037 | 1.060 to 1.205 | <0.001 | 0.860 | 0.015 | 0.831 to 0.889 | <0.001 |
| Practice | P7 | 1.094 | 0.036 | 1.023 to 1.165 | <0.001 | 0.831 | 0.017 | 0.797 to 0.864 | <0.001 |

B is unstandardized; β is standardized. Robust delta-method SEs and 95% CIs are shown on their corresponding scales.

For model identification, the unstandardized loading of one reference indicator per construct (K1, A8, and P1) was fixed at 1.000; therefore, SEs, 95% CIs, and P values are not applicable to these fixed unstandardized parameters. Standardized loadings are reported with their corresponding SEs, 95% CIs, and P values.

**Table S6. KAP score categories**

| Dimension | Possible range | Low cutoff | Low, n (%) | Moderate cutoff | Moderate, n (%) | High cutoff | High, n (%) |
| --- | --- | --- | --- | --- | --- | --- | --- |
| Perceived knowledge | 0–20 | 0–11 | 239 (53.2%) | 12–15 | 70 (15.6%) | 16–20 | 140 (31.2%) |
| Attitude | 8–40 | 8–23 | 67 (14.9%) | 24–31 | 291 (64.8%) | 32–40 | 91 (20.3%) |
| Practice | 7–35 | 7–20 | 106 (23.6%) | 21–27 | 250 (55.7%) | 28–35 | 93 (20.7%) |

Percentages refer to the maximum possible domain score.

**Table S7. Univariable and multivariable regression of perceived knowledge**

| Variable | Univariable B (95% CI) | P | Multivariable B (95% CI) | P |
| --- | --- | --- | --- | --- |
| Sex |  | 0.422 |  |  |
| Male | Reference | — | — | — |
| Female | -0.423 (-1.456 to 0.611) | 0.422 | — | — |
| Age |  | 0.944 |  |  |
| <60 years | Reference | — | — | — |
| ≥60 years | 0.041 (-1.121 to 1.203) | 0.944 | — | — |
| Educational level |  | 0.021 |  |  |
| Junior high school or below | Reference | — | Reference | — |
| High school/technical secondary school | -0.404 (-1.529 to 0.722) | 0.481 | 0.008 (-1.162 to 1.178) | 0.989 |
| Junior college or above | -2.030 (-3.467 to -0.592) | 0.006 | -1.202 (-2.909 to 0.504) | 0.167 |
| Income type |  | <0.001 |  |  |
| No stable income | Reference | — | Reference | — |
| Stable income | 1.929 (0.884 to 2.973) | <0.001 | 1.880 (0.839 to 2.921) | <0.001 |
| Monthly household income per capita |  | 0.042 |  |  |
| <2,000 CNY | Reference | — | Reference | — |
| 2,000–4,999 CNY | -1.357 (-2.755 to 0.041) | 0.057 | -1.355 (-2.706 to -0.003) | 0.049 |
| ≥5,000 CNY | -1.914 (-3.409 to -0.419) | 0.012 | -0.838 (-2.579 to 0.904) | 0.345 |
| Time since prediabetes diagnosis |  | 0.001 |  |  |
| <3 months | Reference | — | Reference | — |
| 3–6 months | 2.833 (1.351 to 4.316) | <0.001 | 2.705 (1.338 to 4.072) | <0.001 |
| >6 months–2 years | 2.104 (0.720 to 3.487) | 0.003 | 1.794 (0.467 to 3.122) | 0.008 |
| >2 years | 1.679 (0.332 to 3.026) | 0.015 | 1.413 (0.113 to 2.712) | 0.033 |
| Hypertension |  | 0.176 |  |  |
| Yes | Reference | — | — | — |
| No | -0.777 (-1.903 to 0.349) | 0.176 | — | — |
| Body mass index |  | 0.962 |  |  |
| <24.0 kg/m² | Reference | — | — | — |
| 24.0–27.9 kg/m² | -0.122 (-1.221 to 0.977) | 0.827 | — | — |
| ≥28.0 kg/m² | -0.193 (-1.852 to 1.465) | 0.819 | — | — |
| Nutrition-therapy education |  | <0.001 |  |  |
| No | Reference | — | Reference | — |
| Yes | 4.716 (3.543 to 5.890) | <0.001 | 4.774 (3.633 to 5.915) | <0.001 |

B, unstandardized OLS coefficient; CI, 95% confidence interval. P values shown on variable-heading rows represent overall univariable tests for the corresponding categorical factor; P values shown for individual categories compare that category with the reference group. Overall univariable P values were used for background-factor selection. Original models and conventional intervals are retained. “—”, not included.

**Table S8. Univariable and multivariable regression of attitude**

| Variable | Univariable B (95% CI) | P | Multivariable B (95% CI) | P |
| --- | --- | --- | --- | --- |
| Sex |  | 0.996 |  |  |
| Male | Reference | — | — | — |
| Female | -0.002 (-0.901 to 0.897) | 0.996 | — | — |
| Age |  | 0.343 |  |  |
| <60 years | Reference | — | — | — |
| ≥60 years | -0.488 (-1.497 to 0.522) | 0.343 | — | — |
| Educational level |  | 0.374 |  |  |
| Junior high school or below | Reference | — | — | — |
| High school/technical secondary school | 0.220 (-0.765 to 1.205) | 0.661 | — | — |
| Junior college or above | -0.709 (-1.967 to 0.549) | 0.268 | — | — |
| Income type |  | 0.018 |  |  |
| No stable income | Reference | — | Reference | — |
| Stable income | 1.103 (0.187 to 2.018) | 0.018 | -0.101 (-0.865 to 0.662) | 0.794 |
| Monthly household income per capita |  | 0.712 |  |  |
| <2,000 CNY | Reference | — | — | — |
| 2,000–4,999 CNY | -0.513 (-1.736 to 0.710) | 0.410 | — | — |
| ≥5,000 CNY | -0.366 (-1.674 to 0.941) | 0.582 | — | — |
| Time since prediabetes diagnosis |  | 0.002 |  |  |
| <3 months | Reference | — | Reference | — |
| 3–6 months | 2.296 (1.006 to 3.586) | <0.001 | 0.682 (-0.325 to 1.689) | 0.184 |
| >6 months–2 years | 1.920 (0.715 to 3.124) | 0.002 | 0.688 (-0.280 to 1.657) | 0.163 |
| >2 years | 1.250 (0.077 to 2.422) | 0.037 | 0.257 (-0.694 to 1.208) | 0.596 |
| Hypertension |  | 0.796 |  |  |
| Yes | Reference | — | — | — |
| No | -0.129 (-1.110 to 0.851) | 0.796 | — | — |
| Body mass index |  | 0.868 |  |  |
| <24.0 kg/m² | Reference | — | — | — |
| 24.0–27.9 kg/m² | -0.057 (-1.012 to 0.898) | 0.907 | — | — |
| ≥28.0 kg/m² | 0.339 (-1.103 to 1.781) | 0.644 | — | — |
| Nutrition-therapy education |  | <0.001 |  |  |
| No | Reference | — | Reference | — |
| Yes | 2.087 (1.016 to 3.159) | <0.001 | -0.639 (-1.532 to 0.254) | 0.160 |
| Perceived knowledge score | 0.569 (0.508 to 0.630) | <0.001 | 0.578 (0.510 to 0.646) | <0.001 |

B, unstandardized OLS coefficient; CI, 95% confidence interval. P values shown on variable-heading rows represent overall univariable tests for the corresponding categorical factor; P values shown for individual categories compare that category with the reference group. Overall univariable P values were used for background-factor selection. Original models and conventional intervals are retained. “—”, not included.

**Table S9. Univariable and multivariable regression of practice**

| Variable | Univariable B (95% CI) | P | Multivariable B (95% CI) | P |
| --- | --- | --- | --- | --- |
| Sex |  | 0.144 |  |  |
| Male | Reference | — | — | — |
| Female | -0.780 (-1.828 to 0.268) | 0.144 | — | — |
| Age |  | 0.004 |  |  |
| <60 years | Reference | — | Reference | — |
| ≥60 years | 1.709 (0.539 to 2.879) | 0.004 | 1.457 (0.529 to 2.385) | 0.002 |
| Educational level |  | 0.329 |  |  |
| Junior high school or below | Reference | — | — | — |
| High school/technical secondary school | -0.650 (-1.801 to 0.500) | 0.267 | — | — |
| Junior college or above | -0.981 (-2.451 to 0.488) | 0.190 | — | — |
| Income type |  | <0.001 |  |  |
| No stable income | Reference | — | Reference | — |
| Stable income | 2.836 (1.792 to 3.879) | <0.001 | 1.603 (0.762 to 2.445) | <0.001 |
| Monthly household income per capita |  | 0.779 |  |  |
| <2,000 CNY | Reference | — | — | — |
| 2,000–4,999 CNY | -0.506 (-1.935 to 0.924) | 0.487 | — | — |
| ≥5,000 CNY | -0.296 (-1.825 to 1.232) | 0.703 | — | — |
| Time since prediabetes diagnosis |  | 0.055 |  |  |
| <3 months | Reference | — | — | — |
| 3–6 months | 2.026 (0.506 to 3.547) | 0.009 | — | — |
| >6 months–2 years | 1.263 (-0.156 to 2.683) | 0.081 | — | — |
| >2 years | 0.636 (-0.746 to 2.017) | 0.366 | — | — |
| Hypertension |  | 0.007 |  |  |
| Yes | Reference | — | Reference | — |
| No | -1.569 (-2.706 to -0.433) | 0.007 | -1.004 (-1.895 to -0.113) | 0.027 |
| Body mass index |  | 0.841 |  |  |
| <24.0 kg/m² | Reference | — | — | — |
| 24.0–27.9 kg/m² | 0.065 (-1.051 to 1.181) | 0.909 | — | — |
| ≥28.0 kg/m² | 0.501 (-1.183 to 2.186) | 0.559 | — | — |
| Nutrition-therapy education |  | 0.917 |  |  |
| No | Reference | — | — | — |
| Yes | 0.068 (-1.205 to 1.340) | 0.917 | — | — |
| Perceived knowledge score | 0.552 (0.473 to 0.631) | <0.001 | 0.258 (0.162 to 0.354) | <0.001 |
| Attitude score | 0.672 (0.583 to 0.761) | <0.001 | 0.464 (0.355 to 0.574) | <0.001 |

B, unstandardized OLS coefficient; CI, 95% confidence interval. P values shown on variable-heading rows represent overall univariable tests for the corresponding categorical factor; P values shown for individual categories compare that category with the reference group. Overall univariable P values were used for background-factor selection. Original models and conventional intervals are retained. “—”, not included.

**Table S10. Diagnostics for the retained original regression models**

| Outcome | Breusch–Pagan P |
| --- | --- |
| Perceived knowledge | 0.039 |
| Attitude | 0.013 |
| Practice | <0.001 |

Heteroscedasticity was detected in all three models.

**Table S11. HC3 sensitivity intervals for the original regression models**

| Outcome | Predictor / contrast | B | HC3 SE | HC3 95% CI | P |
| --- | --- | --- | --- | --- | --- |
| Perceived knowledge | High school vs junior high or below | 0.008 | 0.606 | -1.182 to 1.198 | 0.989 |
| Perceived knowledge | College or above vs junior high or below | -1.202 | 0.872 | -2.916 to 0.512 | 0.169 |
| Perceived knowledge | Stable vs no stable income | 1.880 | 0.501 | 0.895 to 2.866 | <0.001 |
| Perceived knowledge | Income 2,000–4,999 vs <2,000 CNY | -1.355 | 0.651 | -2.635 to -0.075 | 0.038 |
| Perceived knowledge | Income ≥5,000 vs <2,000 CNY | -0.838 | 0.858 | -2.524 to 0.849 | 0.330 |
| Perceived knowledge | Diagnosis 3–6 vs <3 months | 2.705 | 0.665 | 1.399 to 4.011 | <0.001 |
| Perceived knowledge | Diagnosis >6 months–2 years vs <3 months | 1.794 | 0.672 | 0.474 to 3.115 | 0.008 |
| Perceived knowledge | Diagnosis >2 years vs <3 months | 1.413 | 0.587 | 0.259 to 2.566 | 0.016 |
| Perceived knowledge | Nutrition-therapy education: yes vs no | 4.774 | 0.569 | 3.655 to 5.892 | <0.001 |
| Attitude | Stable vs no stable income | -0.101 | 0.397 | -0.881 to 0.679 | 0.799 |
| Attitude | Diagnosis 3–6 vs <3 months | 0.682 | 0.544 | -0.388 to 1.752 | 0.211 |
| Attitude | Diagnosis >6 months–2 years vs <3 months | 0.688 | 0.483 | -0.261 to 1.637 | 0.155 |
| Attitude | Diagnosis >2 years vs <3 months | 0.257 | 0.489 | -0.705 to 1.219 | 0.600 |
| Attitude | Nutrition-therapy education: yes vs no | -0.639 | 0.449 | -1.522 to 0.244 | 0.156 |
| Attitude | Perceived knowledge | 0.578 | 0.038 | 0.504 to 0.652 | <0.001 |
| Practice | Age ≥60 vs <60 years | 1.457 | 0.495 | 0.485 to 2.429 | 0.003 |
| Practice | Stable vs no stable income | 1.603 | 0.439 | 0.740 to 2.466 | <0.001 |
| Practice | No hypertension vs hypertension | -1.004 | 0.466 | -1.920 to -0.088 | 0.032 |
| Practice | Perceived knowledge | 0.258 | 0.055 | 0.150 to 0.366 | <0.001 |
| Practice | Attitude | 0.464 | 0.065 | 0.337 to 0.591 | <0.001 |

Same observations, predictors and OLS coefficients as Tables S7–S9. Only covariance estimation is changed to HC3, with residual-df t inference. HC3 standard errors were used to provide heteroscedasticity-robust inference.

**Table S12. Spearman correlations among KAP scores**

| Spearman | Perceived knowledge | Attitude | Practice |
| --- | --- | --- | --- |
| Perceived knowledge | 1.000 |  |  |
| Attitude | 0.617 (P<0.001) | 1.000 |  |
| Practice | 0.427 (P<0.001) | 0.494 (P<0.001) | 1.000 |

**Table S13. SEM fit indices**

| Index | Scaled | Robust |
| --- | --- | --- |
| Scaled χ² / df | 699.513 / 266 | — |
| CFI | 0.985 | 0.902 |
| TLI | 0.983 | 0.889 |
| RMSEA (90% CI) | 0.060 (0.055 to 0.066) | 0.096 (0.089 to 0.103) |
| SRMR | 0.041 | — |

The CFA and SEM use the same measurement structure and six residual covariances.

Abbreviations: χ², chi-square; df, degrees of freedom; CFI, comparative fit index; TLI, Tucker–Lewis index; RMSEA, root mean square error of approximation; CI, confidence interval; SRMR, standardized root mean square residual.

**Table S14. Indirect and total associations in the primary SEM**

| Association K–P | Scale | Estimate | Delta SE | Delta P | Monte Carlo 95% CI |
| --- | --- | --- | --- | --- | --- |
| Indirect | Unstandardized B | 0.338 | 0.048 | <0.001 | 0.246 to 0.435 |
| Indirect | Standardized β | 0.349 | 0.048 | <0.001 | 0.257 to 0.444 |
| Total | Unstandardized B | 0.557 | 0.037 | <0.001 | 0.485 to 0.629 |
| Total | Standardized β | 0.575 | 0.029 | <0.001 | 0.518 to 0.631 |

Indirect association is K→A→P; total association is the direct K→P coefficient plus the indirect association. All estimates derive from the primary WLSMV model with six prespecified residual covariances. Monte Carlo intervals use 100,000 joint parameter draws; SE and P columns use the robust delta method and are labeled separately.
