# Supplementary material for: Perceived knowledge, attitudes, and practices regarding nutrition therapy among people with prediabetes: a structural equation model analysis
Source: Front Nutr. 2026 Oct 29;13:1952271. doi: 10.3389/fnut.2026.1952271 (PMC13754649; doi:10.3389/fnut.2026.1952271)
Supplement: Supplementary file 2 [file Table_2.docx]

# Participant information and consent

Dear Participant,

Hello! We sincerely invite you to take part in this questionnaire survey. This study aims to understand the knowledge, attitudes, and practices regarding nutrition therapy among individuals with prediabetes, with the goal of providing evidence for the development of scientifically informed intervention strategies in the future.

This study has received ethical approval. Your participation is entirely voluntary. There are no right or wrong answers to the questionnaire, and you are kindly asked to respond according to your actual situation. The survey only involves basic personal information and health-related knowledge and perceptions. We will strictly protect your privacy, and all collected information will be used solely for scientific research and analysis. Your personal information will not be disclosed. You may obtain information related to the study at any time and may withdraw from the study at any stage. If you withdraw, your data will not be included in the analysis.

We sincerely thank you for your support of this study.

□ I have been informed about the study and agree that the collected data may be used for scientific research.

# Part 1 General characteristics

Sex: Male; female

Age: Years (integer)

Residence: Urban; rural

Education: Junior high school or below; high school/technical secondary school; junior college; bachelor’s degree or above

Marital status: Never married; married; divorced; widowed

Income type: No stable income; stable income

Monthly household income per capita: <2,000; 2,000–5,000; 5,000–10,000; 10,000–20,000; >20,000 CNY (categories as worded in the source questionnaire)

Time since being told you have prediabetes: Within 3 months; 3–6 months; 6 months–2 years; more than 2 years

Hypertension: Yes; no

Height: cm (integer)

Weight: kg (integer)

Previous nutrition-therapy education from a hospital or other medical institution: No; yes

Usual sources of dietary health information (multiple choices permitted): Hospital or medical professionals; relatives/friends; news/television; social media

# Part 2 Perceived knowledge

For each statement, indicate your familiarity: very familiar (2); heard of it (1); do not know (0).

K1. Prediabetes is a condition between normal blood glucose and diabetes.

K2. Nutrition therapy and lifestyle intervention during prediabetes can delay or prevent diabetes.

K3. Dietary fiber from whole grains, legumes, and vegetables can slow glucose elevation and improve insulin sensitivity.

K4. People with prediabetes need to control total energy intake.

K5. Regular meals with consistent timing and portions are more beneficial for glycemic control.

K6. People with prediabetes who are overweight or obese need to prioritize weight management.

K7. Excessive intake of refined sugar and sugar-sweetened beverages is unfavorable for prediabetes control.

K8. High-glycemic-index foods such as white bread and sugar-sweetened beverages rapidly increase postprandial glucose.

K9. People with prediabetes should combine dietary intervention with physical activity.

K10. Eating vegetables first, protein foods second, and staple foods last can reduce postprandial glucose responses.

Attention check (not scored in KAP): “Among the 56 ethnic groups in China, the Han population is the smallest.” Response options: true; false; do not know. The correct response is false.

# Part 3 Attitudes

Strongly agree (5); agree (4); neutral (3); disagree (2); strongly disagree (1).

A1. I believe that intervention should begin early during the prediabetes stage.

A2. I believe that nutrition therapy is important for delaying or preventing diabetes.

A3. I am confident that I can adhere to a healthy eating plan.

A4. I believe that nutrition therapy can effectively control or reverse prediabetes and prevent progression to diabetes.

A5. I am willing to adjust my diet under the guidance of a physician or dietitian.

A6. Compared with medication, I prefer to manage blood glucose through diet and lifestyle changes.

A7. I believe that dietary control during prediabetes is more meaningful than waiting to treat diabetes after it develops.

A8. I believe that combining exercise with diet produces better results.

# Part 4 Practices

Please answer for the past 3 months. Always (5): almost 100%; often (4): approximately more than 70%; sometimes (3): approximately 40–70%; rarely (2): approximately 10–40%; never (1): never or below 10%. These anchors reproduce the source wording.

P1. I actively seek dietary information suitable for prediabetes.

P2. I measure my blood glucose regularly.

P3. I control my daily food intake according to my own condition.

P4. I consciously control staple-food portions and combine refined and whole-grain staple foods.

P5. I check nutrition information on food packaging.

P6. I eat vegetables first, meat or eggs second, and staple foods last.

P7. I record weight changes or pay attention to weight management.
